# Supplementary figures and images for: Long noncoding RNAs emerge from transposon-derived antisense sequences and may contribute to infection stage-specific transposon regulation in a fungal phytopathogen
Source: Mob DNA. 2023 Nov 15;14:17. doi: 10.1186/s13100-023-00305-6 (PMC10648671; doi:10.1186/s13100-023-00305-6)

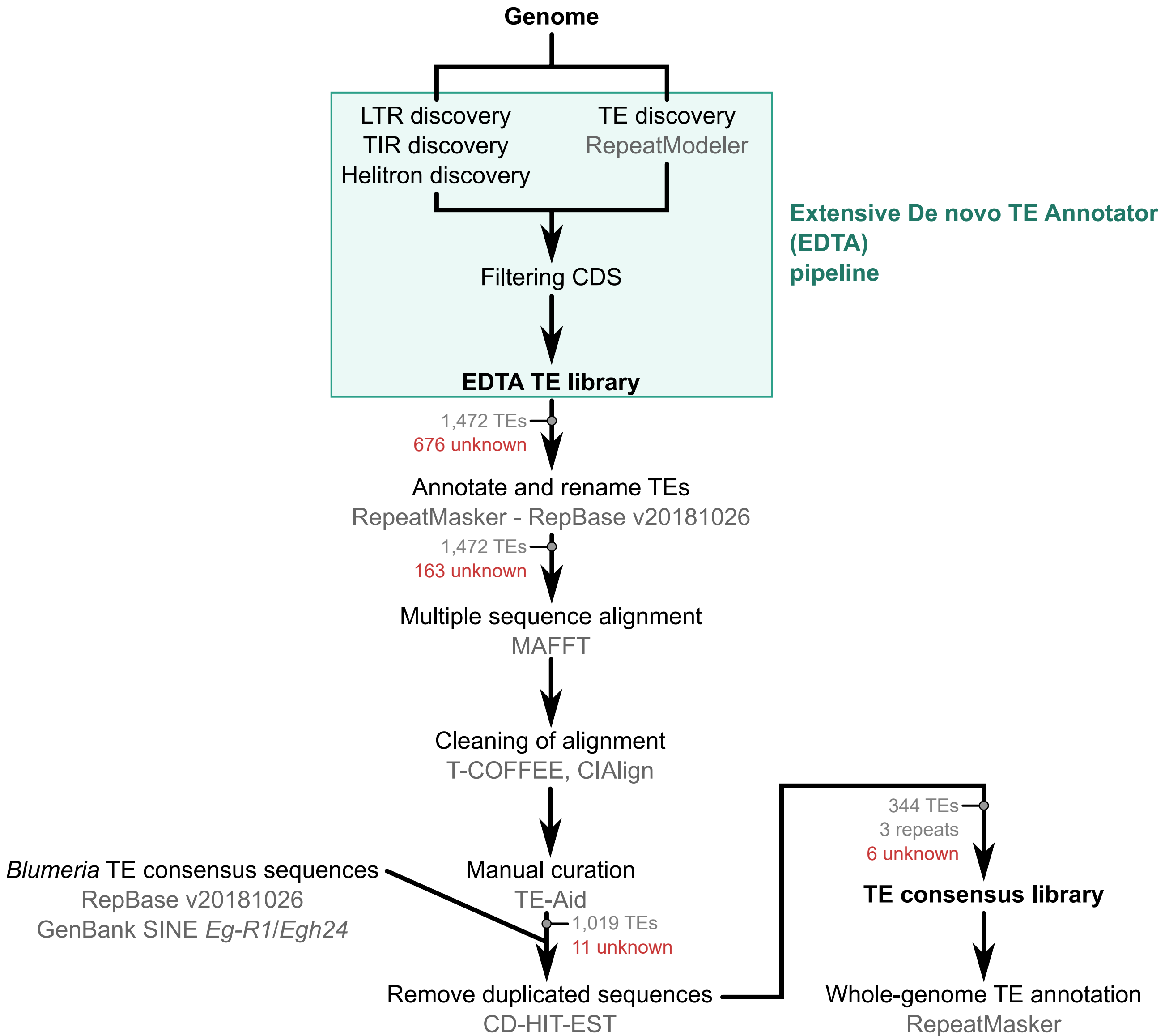

Supplement: Supplementary file 1 — Additional file 1: Supplementary Fig. 1. Integrated pipeline for high-quality genome-wide TE annotation. We initially detected transposable elements (TEs) the genome assembly of B. hordei isolate DH14 [10] using the extensive de novo annotator (EDTA) pipeline [90], which enables de novo discovery of long terminal repeat (LTR), terminal inverted repeat (TIR), DNA helitron, and long interspersed nuclear element (LINE) TEs. We performed an initial curation of the database using the Blumeria TE consensus sequences from RepBase v20181026 via RepeatMasker [91]. Then, we manually curated the TE consensus database, as described in [35]. Briefly, we generated the consensus repeat database via multiple sequence alignment with MAFFT v7.475 [92] and clustered sequences, removed gaps, and deleted divergent sites using T-COFFEE v11.00.8cbe486 [93] and CIAlign v1.0.18 [94]. We manually adjusted overhangs to define TE boundaries with the help of AliView [95] and TE-Aid v.0-dev [92]. Blumeria TE consensus sequences from RepBase v20181026 and short interspersed nuclear element (SINE) TE sequences extracted from GenBank accessions X86077.1 (Eg-R1) and Z21962.1 (Egh24) were merged with the manually curated database and duplicated sequences subsequently eliminated with CD-HIT-EST [96]. Genome-wide re-annotation of TEs in the genome assembly of B. hordei isolate DH14 [10] was done with RepeatMasker [91] using the manually curated repeat consensus library as database (Table 1; Supplementary Table 2). The number of annotated TEs at various stages of the pipeline is indicated in grey and the number of TEs without an annotation, denoted as unknown, is shown in red. [file 13100_2023_305_MOESM1_ESM.pdf]

Relative transcript abundance

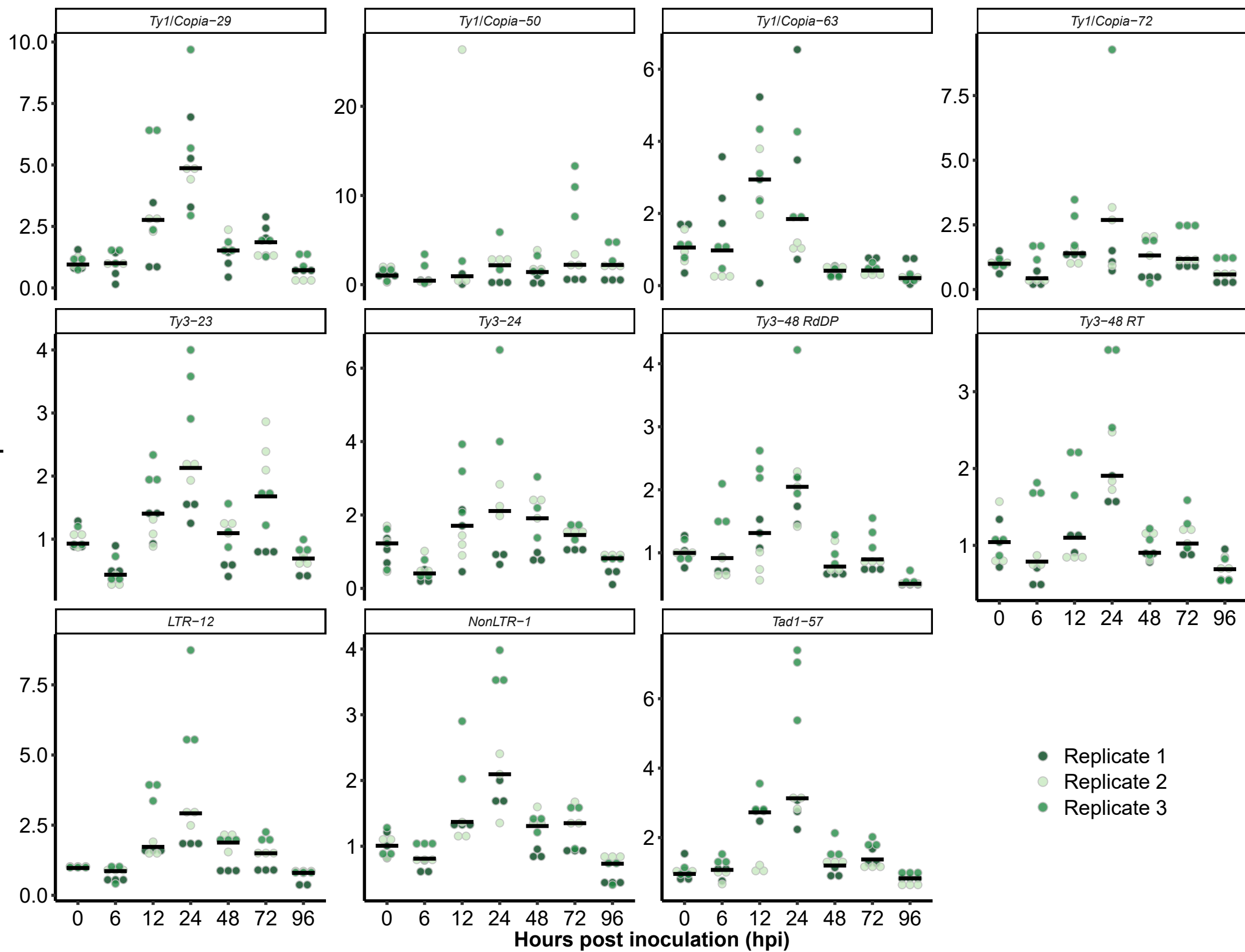

Supplement: Supplementary file 2 — Additional file 2: Supplementary Fig. 2. B. hordei TEs exhibit time point-specific upregulation during host infection. We performed quantitative reverse transcriptase-polymerase chain reaction (qRT-PCR) analysis of eleven selected TE families. The dot plots show the relative transcript abundance (according to ΔΔCT analysis; y-axis) of the respective TE family, indicated on the top of each panel, in B. hordei isolate K1AC at seven time points of host infection (hours post inoculation (hpi); x-axis). TE transcript levels were normalized to B. hordei GAPDH (BLGH_00691); we conducted three independent replicates (n = 3) consisting of three technical replicates each. The shades of green indicate the replicate each data point belongs to; the black bar shows the median. [file 13100_2023_305_MOESM2_ESM.pdf]

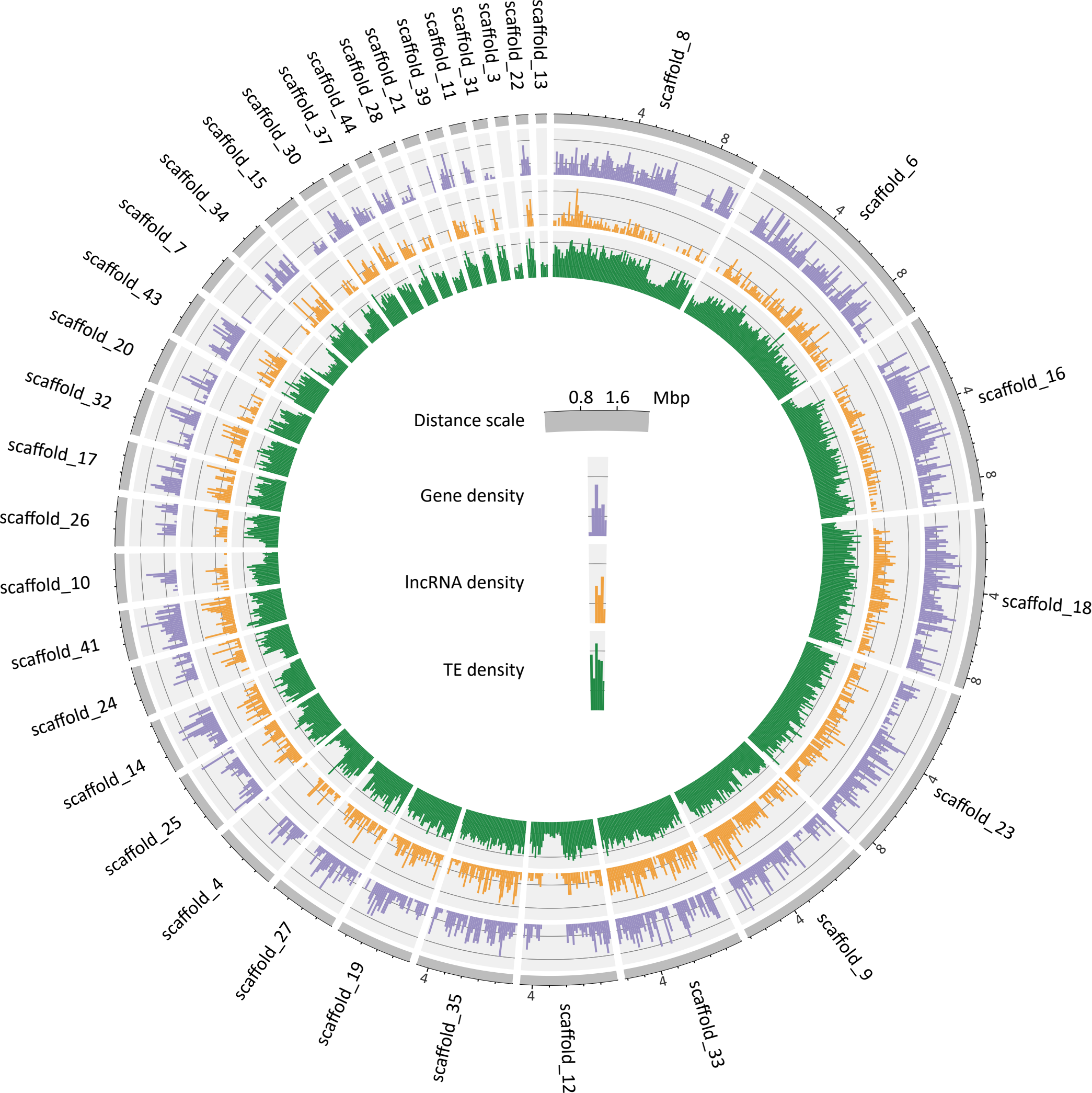

Supplement: Supplementary file 3 — Additional file 3: Supplementary Fig. 3. B. hordei lncRNAs are ubiquitously distributed throughout the genome. The circos plot displays the density of coding genes (purple), lncRNAs (orange), and TEs (green) on all scaffolds larger than 500 kb in the genome assembly of B. hordei DH14 [10]. We generated density maps of B. hordei DH14 coding genes, lncRNAs, and TEs in 100-kb windows using BEDtools v2.25.0 [97]; these windows are plotted as bar plots in the three tracks. The data was visualized using the R package Rcircos v1.2.2 [98]. [file 13100_2023_305_MOESM3_ESM.pdf]

**A**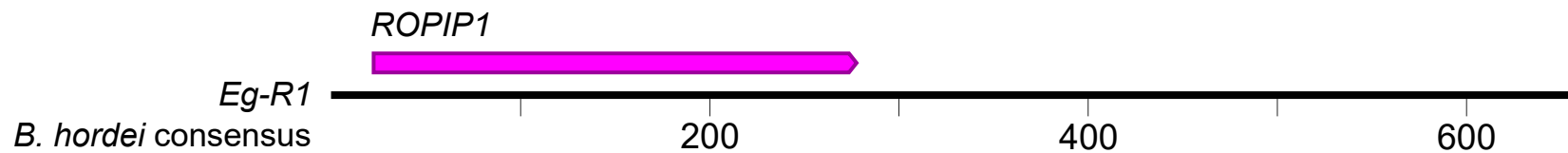**B**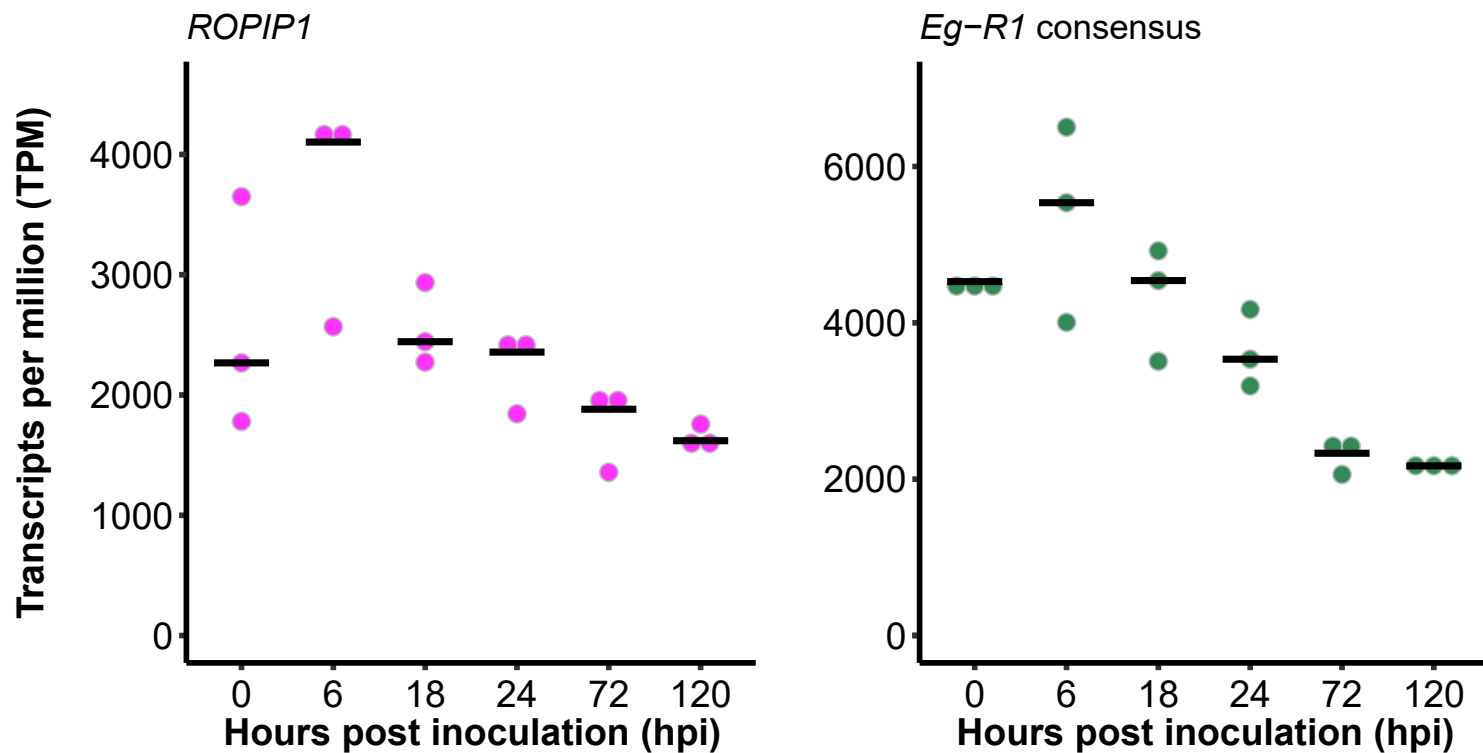

Supplement: Supplementary file 4 — Additional file 4: Supplementary Fig. 4. B. hordei ROPIP1 expression peaks in appressoria prior to haustorium formation. A The ROPIP1 gene, which encodes a host-translocated peptide that disturbs barley microtubules [11], locates to the Eg-R1 consensus sequence (654 bp in length). The upper lane displays the annotated ROPIP1 transcript on Eg-R1, indicated in magenta. The scale below the black horizontal line indicates the TE length and position in bp. B The ROPIP1 and Eg-R1 expression patterns throughout the infection cycle of B. hordei is shown as dot plots, as indicated on top of each plot. Expression values are indicated as transcripts per million (TPM; y-axis) during six time points of host infection (x-axis; see Fig. 1A). The black bar shows the median of three independent replicates (n = 3). [file 13100_2023_305_MOESM4_ESM.pdf]

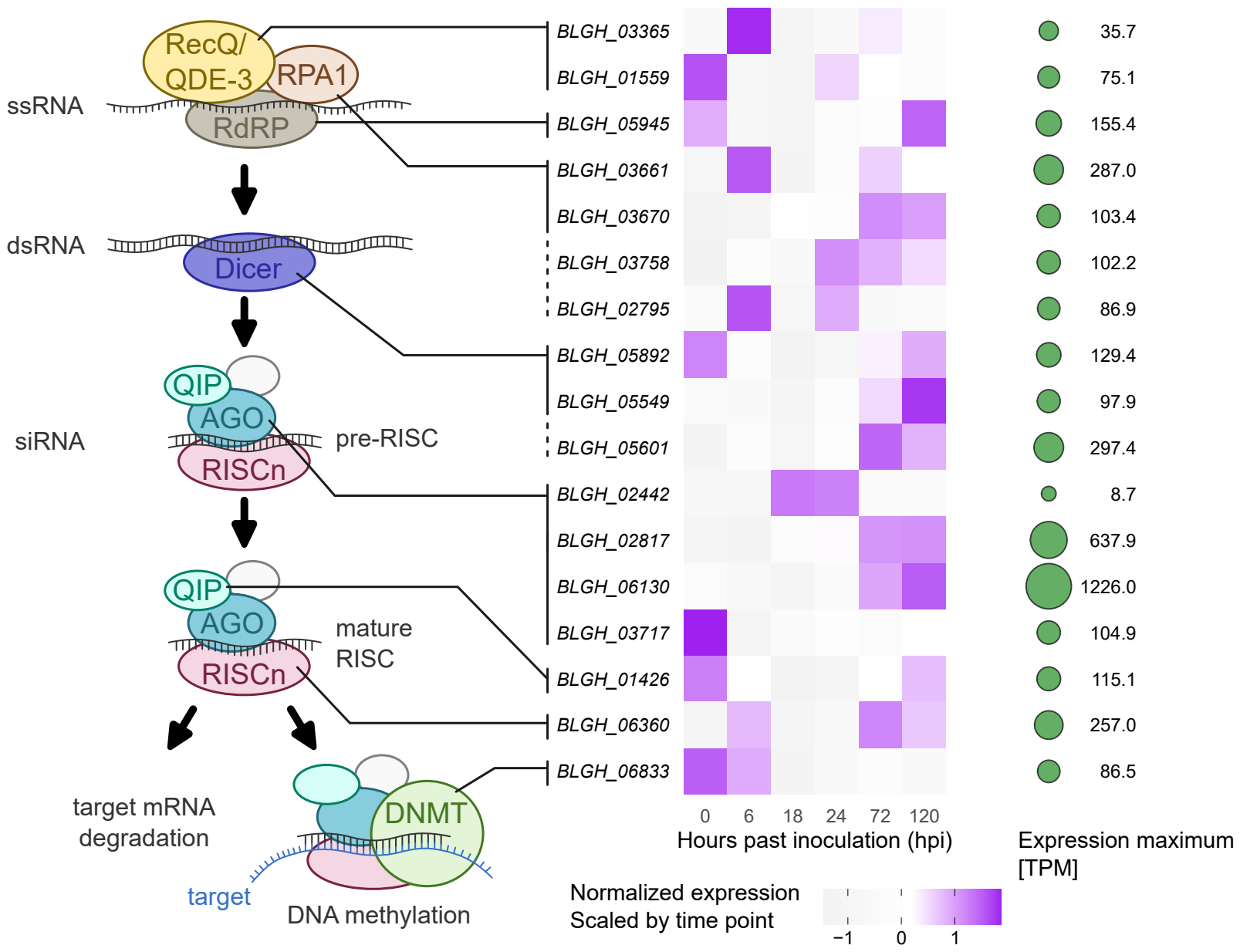

Supplement: Supplementary file 5 — Additional file 5: Supplementary Fig. 5. Components of the RNAi machinery in B. hordei are expressed in a time point-dependent manner. The left panel shows the simplified canonical RNAi pathway, excluding microRNA biogenesis. Briefly, the RNA-dependent RNA polymerase (RdRP) binds single-stranded RNA (ssRNA) and associates with a helicase (quelling-deficient 3; QDE-3) and replication factor A1 (RPA1). Upon synthesis of the complementary strand to generate double-stranded RNA (dsRNA), Dicer binds and generates 20–30 nucleotides-long short interfering RNAs (siRNAs). Subsequently, AGO (syn. QDE-2 in Neurospora crassa), the AGO-binding protein QDE-2 interacting protein (QIP) and the RISC nuclease (RISCn) are recruited to form the pre-RNA-induced silencing complex (RISC). The mature RISC is formed upon release of the complementary strand, leaving the target siRNA strand, which facilitates complementary binding to target RNA molecules. The RISC complex then catalyzes target mRNA degradation or RNAi-directed DNA methylation by recruitment of DNA methylase (DNMT). The respective B. hordei orthologs are assigned on the right panel; dotted lines indicate B. hordei paralogs of RPA2 (BLGH_03758) and RPA3 (BLGH_02795) and a Piwi domain-containing protein with similarity to Dicer (BLGH_05601) whose homology and role in RNAi are unclear [16]. The right panel shows a heatmap of normalized and time point-scaled relative expression of the ortholog genes encoding RNAi components in B. hordei; purple indicates high relative expression compared to average throughout the time points. The maximum expression throughout all time points in transcripts per million (TPM) is shown on the right with green circles; the respective TPM values are indicated on the right from the circles. [file 13100_2023_305_MOESM5_ESM.pdf]

**A**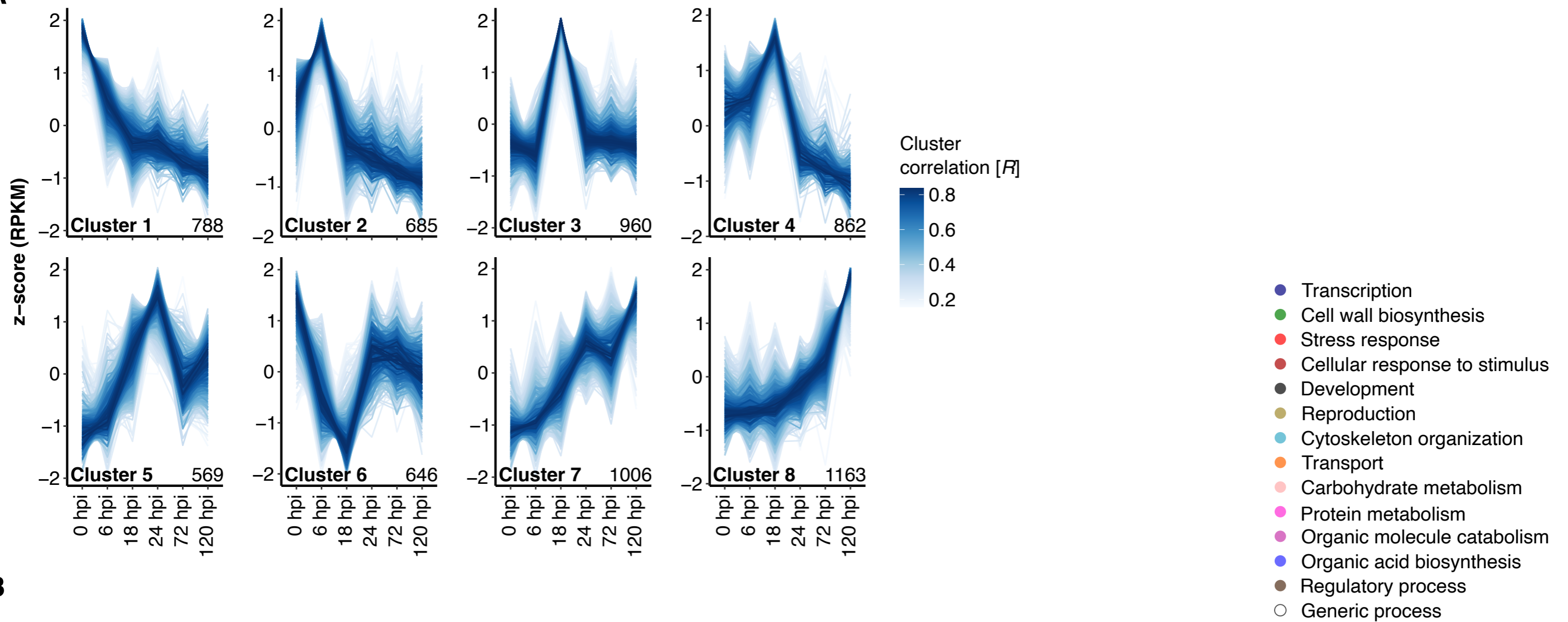**B**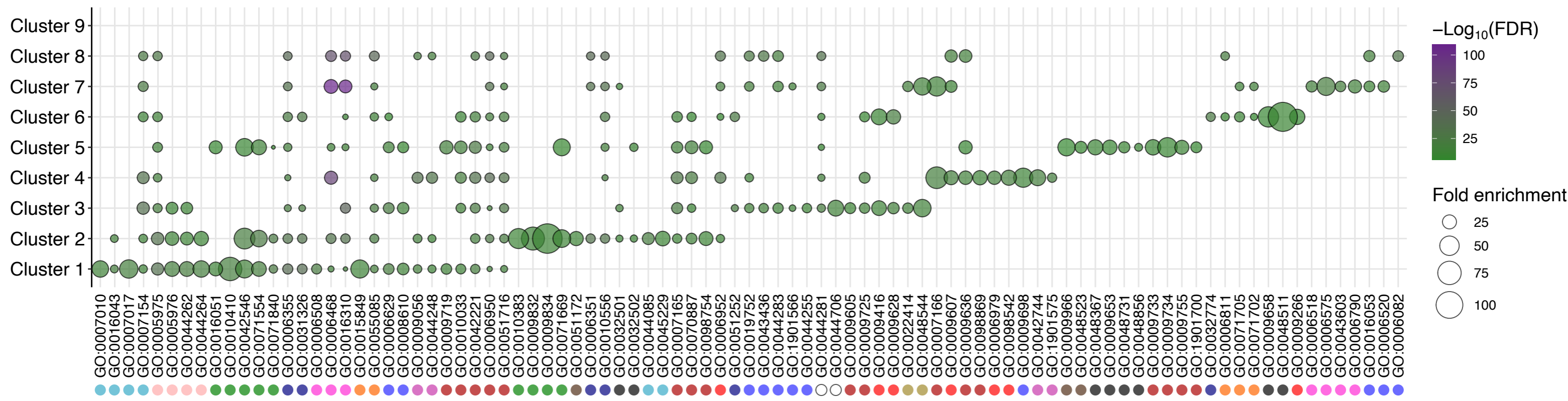

Supplement: Supplementary file 6 — Additional file 6: Supplementary Fig. 6. H. vulgare displays infection stage-dependent expression patterns of coding genes upon infection with B. hordei. A We clustered time-resolved coding gene expression patterns in the host H. vulgare cv. ‘Margret’ upon infection with B. hordei K1AC using TCseq [37]. The RNA-seq data was mapped to the H. vulgare cv. ‘Morex’ reference genome [34] using HISAT2 [45] with ‘–max-intronlen 500’ and parsed output using SAMtools v1.9 [104] and BEDtools v2.25.0 [97]. The lines represent single transcripts; the color shade denotes the cluster membership by Spearman correlation (the darker the shade of blue, the higher the correlation R with the respective expression cluster according to the color scheme). The numbers at the bottom-right of each plot indicates the number of genes that made up each of the time course clusters. The x-axis denotes the time points of infection in hours post inoculation (hpi; Fig. 1A), which were 0 hpi (conidiospore germination), 6 hpi (appressorium formation), 18 hpi (host cell penetration), 24 hpi (haustorium formation), 72 hpi (epiphytic colonization), and 120 hpi (conidiogenesis). The y-axis indicates the relative z-score based on reads per kb of transcript per million mapped reads (RPKM). B We performed gene ontology (GO) enrichment analysis for the H. vulgare coding genes from each cluster (A) using ShinyGO v0.77 [51] accessed online at http://bioinformatics.sdstate.edu/go/, and summarized GO terms with REVIGO [52]. The dot plot displays the fold enrichment of functional terms compared to the full set of genes of H. vulgare cv. ‘Morex’ (dot size); the fill color indicates the − Log10(FDR-adjusted enrichment p value) according to the color scheme on the right. The GO term accessions are indicated on the x-axis; colored dots denote summarizing GO descriptions indicated in the legend (top-right). The respective co-expression cluster (A) is indicated on the y-axis. [file 13100_2023_305_MOESM6_ESM.pdf]

**A**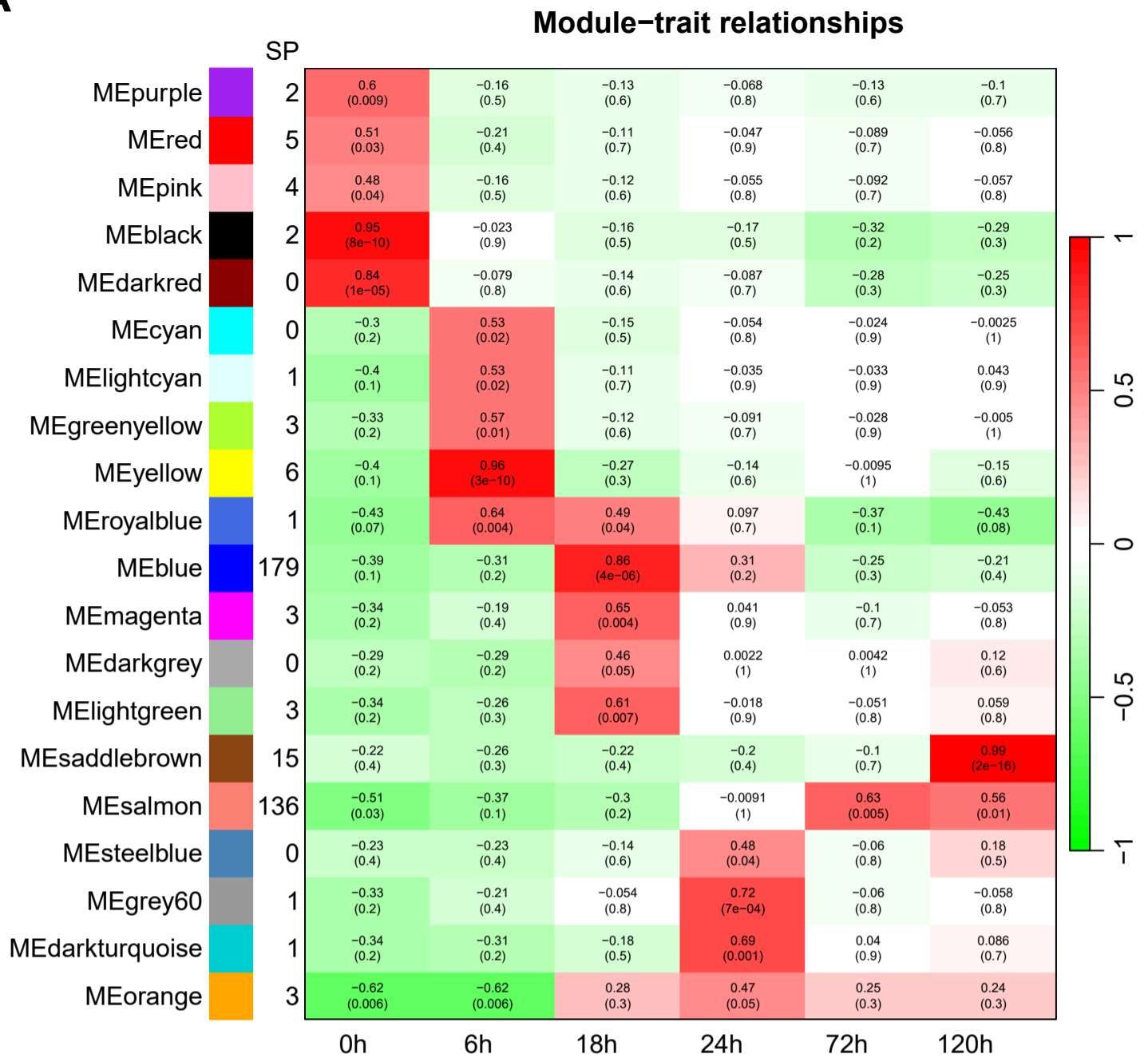**B**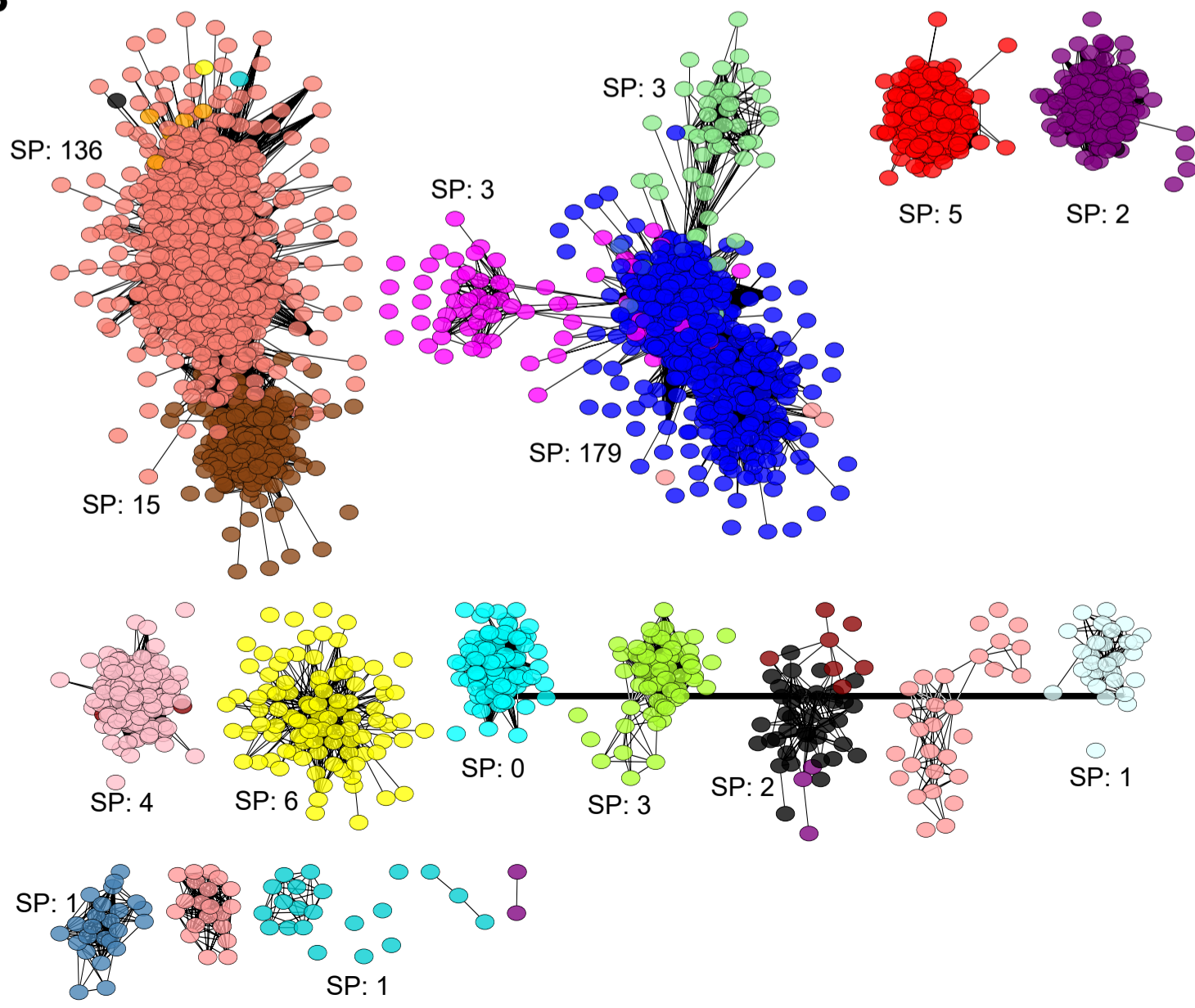

Supplement: Supplementary file 7 — Additional file 7: Supplementary Fig. 7. Co-expression analysis of TEs, coding genes, and lncRNAs in B. hordei. A We built co-regulation networks using WGCNA [55] using the time course expression data of B. hordei coding genes (mRNAs), lncRNAs, and consensus TEs. The time points of infection were 0 hpi (spore germination), 6 hpi (appressorium formation), 18 hpi (early primary haustorium), 24 hpi (mature primary haustorium), 72 hpi (host colonization), and 120 hpi (conidia formation). The twenty identified co-expression clusters are indicated on the y-axis and the time points on the x-axis. The column SP indicates the number of genes encoding putative secreted proteins (SPs) in each cluster. The Pearson correlation coefficient of each cluster with the respective time point is shown on a scale from green to red, indicating negative to positive correlation. The exact Pearson correlation value and the p value are shown for each cluster and time point. B The colored circles indicate transcripts and lines significant correlation between two transcripts. The circles are colored according to the WGCNA-assigned cluster colors (A); SP indicates the number of genes encoding putative secreted proteins in the respective co-expression cluster. The clusters correspond to the clusters in Fig. 6. [file 13100_2023_305_MOESM7_ESM.pdf]
